# MiR-302c inhibits tumor growth of hepatocellular carcinoma by suppressing the endothelial-mesenchymal transition of endothelial cells

**DOI:** 10.1038/srep05524

**Published:** 2014-07-16

**Authors:** Kai Zhu, Qi Pan, Luo-qi Jia, Zhi Dai, Ai-wu Ke, Hai-ying Zeng, Zhao-you Tang, Jia Fan, Jian Zhou

**Affiliations:** 1 Liver Cancer Institute, Zhong Shan Hospital, Fudan University, Shanghai, 200032 China; Key Laboratory of Carcinogenesis and Cancer Invasion (Fudan University), Ministry of Education; 2 Department of Gynecology, Obstetrics and Gynecology Hospital, Shanghai Key Laboratory of Female Reproductive Endocrine Related Diseases, Fudan University, Shanghai 200032, China; 3 Department of Pathology, Zhong Shan Hospital, Fudan University, Shanghai 200032, China; 4 Shanghai Key Laboratory of Organ Transplantation, Zhong Shan Hospital, Fudan University, Shanghai 200032, China; 5 These authors contributed equally to this work.

## Abstract

Endothelial cells (ECs) are critical for angiogenesis, and microRNA plays important roles in this process. In this study, we investigated the function and mechanism of miR-302c in the process of endothelial-mesenchymal transition (EndMT) in ECs. When miR-302c was overexpressed in HUVECs, the motility of the HUVECs was weakened; the expression levels of EndMT markers were also changed: vascular endothelial (VE)-cadherin was up-regulated, whereas β-catenin, FSP1, and α-SMA were down-regulated. Further *in vivo* and *in vitro* experiments showed that the growth of HCC was inhibited when co-cultured or co-injected with HUVECs overexpressing miR-302c. On the contrary, when miR-302c was suppressed in HUVECs, the opposite results were observed. Reporter assays showed that miR-302c inhibited metadherin (MTDH) expression through directly binding to its 3′UTR. In addition, compared to ECs isolated from normal liver tissues of HCC patients, ECs isolated from tumor tissues expressed markedly low levels of miR-302c but high levels of MTDH. These results suggest that EC-specific miR-302c suppresses tumor growth in HCC through MTDH-mediated inhibition of EndMT. MTDH and miR-302c might provide a new strategy for anti-angiogenic therapy in HCC.

Tumor growth relies not only on the malignancy of the tumor cells but also on the interaction between tumor cells and stromal cells^1^. Tumor stromal cells consist of various types of cells, including cells of the vascular and immune systems, fibroblasts, etc.^2^ The interactions between tumor cells and stromal cells occur in two ways: tumor cells can activate stromal cells in a paracrine fashion, and the activated stromal cells may have either tumor-promoting or tumor–suppressing functions.

The tumor vascular system, including endothelial cells (ECs), smooth muscle cells and pericytes, is crucial in the activation of angiogenesis and the progression of tumors^3^,^4^. Recent studies have indicated that ECs contribute to cancer progression through the endothelial-to-mesenchymal transition (EndMT)^5^,^6^,^7^. EndMT is a process whereby ECs acquire a mesenchymal phenotype characterized by loss of cell–cell junctions and endothelial markers (e.g., VE-cadherin, CD31), as well as the gain of invasive and migratory properties and mesenchymal markers (e.g., FSP-1, α-SMA)^8^. EndMT was first observed in the embryonic development of the heart^9^, and recent studies suggest that EndMT occurs in a variety of pathological processes, including cardiac fibrosis^5^, chronic pulmonary hypertension^10^ and cancer^5^,^6^,^11^.

Increasing evidence has indicated that microRNAs play important roles in regulating EC activity and angiogenesis^12^,^13^,^14^. Our previous study compared the microRNA expression profile of human umbilical vein endothelial cells (HUVECs) in the absence or presence of hepatocellular carcinoma (HCC) cells^14^ and found that 4 microRNAs either increased or decreased by more than 1.5-fold, among which miR-302c was the most highly down-regulated microRNA in HUVECs exposed to HCC cells. In this study, we investigated the function of miR-302c in the process of EndMT in ECs.

## Results

### MiR-302c inhibits the motility of HUVECs

**Figure 1:**
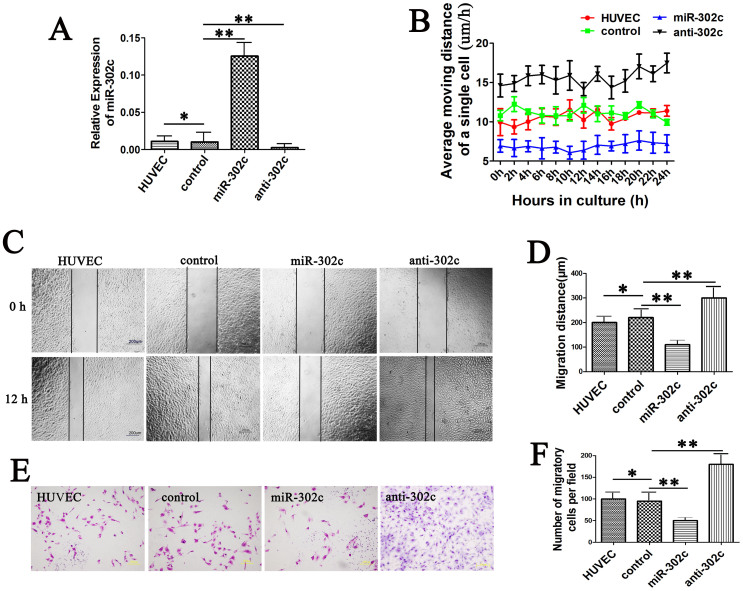
Effect of miR-302c on the motility of HUVECs. (A) RT-PCR results of miR-302c expression in untreated HUVECs or HUVECs transfected with lentivirus; U6 was used as an internal control. (B) Motility of HUVECs assessed by the average moving distance of a single cell. (C) Images of scratch assays in HUVECs. (D) Quantification of the migration distances shown in C. (E) Images of the two-chamber migration assay. (F) Quantification of the number of migratory cells shown in E. (*P > 0.05, **P < 0.05).

To explore the role of miR-302c in ECs, we stably overexpressed or suppressed miR-302c in HUVECs (Fig. 1A). We investigated the effect of miR-302c on the motility of HUVECs by monitoring the cells in the Cell-IQ system for 4 days. The results showed that overexpression of miR-302c in HUVECs led to a marked decrease in the motility (average moving distance: 7 vs. 11 μm/h, *P* < 0.05); on the contrary, when miR-302c was suppressed in HUVECs, the opposite results were observed (average moving distance: 15.6 vs. 11 μm/h, *P* < 0.05) (Fig. 1B). We also performed scratch assay (Fig. 1C, D) and two-chamber migration assay (Fig. 1E, F) to assess the horizontal and vertical migratory abilities of the cells; the results confirmed that miR-302c can inhibit the migratory ability of HUVECs.

### MiR-302c inhibits the EndMT process of HUVECs

**Figure 2:**
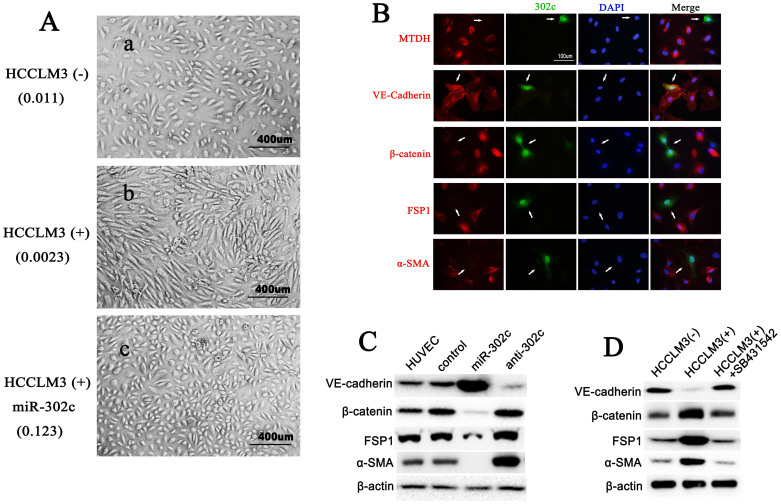
MiR-302c inhibits EndMT of HUVECs. (A) Representative images of HUVECs under different conditions; a, HUVECs in the absence of HCCLM3 cells; b, HUVECs in the presence of HCCLM3 cells; c, HUVECs overexpressing high level of miR-302c, and in the presence of HCCLM3 cells. In the parentheses are the relative levels of miR-302c of the HUVECs. (B) Immunofluorescence images of MTDH and EndMT markers (VE-cadherin, β-catenin, FSP1 and α-SMA) (red) in HUVECs transfected with the lentiviral vector pSicoR-miR-302c (green). Arrowed are cells expressing high levels of miR-302c. (C) Western blot analysis of EndMT markers in HUVECs harboring different levels of miR-302c. (D) Western blot analysis of EndMT markers in HUVECs under different growth conditions.

When we co-cultured HUVECs with HCCLM3 cells, we observed that, compared to HUVECs cultured alone (Fig. 2Aa), HUVECs exposed to HCCLM3 cells lost their endothelial morphology and gained a mesenchymal type (Fig. 2Ab). However, when HUVECs were transfected with viral particles to overexpress miR-302c, they retained an endothelial shape, even in the presence of HCCLM3 cells (Fig. 2Ac). This result suggests that miR-302c inhibits the EndMT of HUVECs.

To validate our hypothesis, we performed an immunofluorescence microscopy assay of EndMT markers (VE-cadherin, β-catenin, FSP1 and α-SMA) on the HUVECs transfected with miR-302c-overexpressing virus (lentiviral GFP vector pSicoR-miR-302c). Forty-eight hours after transfection, 20% of the cells expressed a higher level of miR-302c, as shown by the existence of green fluorescence (Fig. 2B). These HUVECs exhibited a more “endothelial-shaped” phenotype compared to those without green fluorescence, i.e., those expressing lower levels of miR-302c. The results of the immunofluorescence assay showed that overexpressing miR-302c in HUVECs led to markedly increased VE-cadherin expression and decreased β-catenin, FSP1 and α-SMA expression levels. Similar results were observed in western blot assays of the expression of EndMT markers in HUVECs (Fig. 2C). These results confirmed that miR-302c can inhibit the EndMT of ECs.

It is not clear how the co-culturing of HCCLM3 cells induces the EndMT of HUVECs. Studies have shown that EndMT can be induced by various cytokines including TGF-β^5^,^8^,^15^. In order to examine whether HCCLM3 cells induce the EndMT of HUVECs through secreting TGF-β, we blocked TGF-β signal of HUVECs by adding a TGF-β receptor kinase inhitor (SB431542, ab120163, Abcam) in the co-culturing system. Western blot of the EndMT markers showed that HCCLM3 induces the EndMT of HUVECs, but when SB431542 was added to block TGF-β signal of HUVECs, the EndMT was reversed (Fig. 2D). The results suggest that HCCLM3 cells might induce the EndMT of HUVECs through secreting TGF-β.

### Overexpression of miR-302c in HUVECs inhibits growth of co-cultured HCCLM3 cells

**Figure 3:**
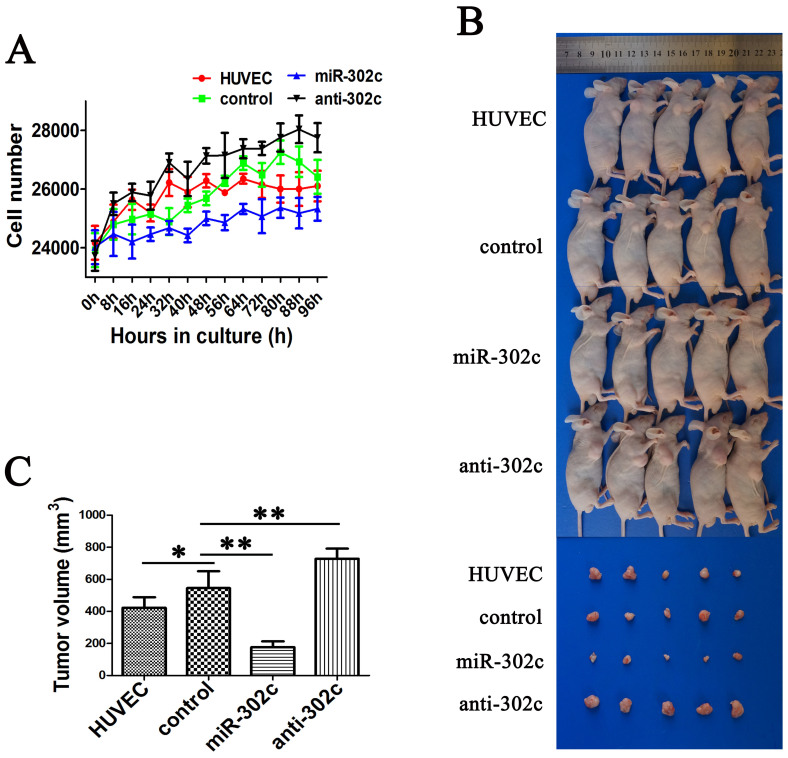
Overexpression of miR-302c in HUVECs inhibits the growth of co-cultured HCCLM3 cells. (A) Proliferation rate of HCCLM3 cells exposed to HUVECs with different levels of miR-302c assessed by growth curves of cells in a 24-well plate. (B) Macroscopic tumors formed by co-injecting HCCLM3 cells and HUVECs (harboring either gain or loss of miR-302c) in the right flanks of nude mice. (C) Quantification of the tumor volume. (*P > 0.05, **P < 0.05).

Because ECs may contribute to cancer progression through EndMT^5^,^6^,^11^, we sought to determine whether miR-302c-mediated EndMT in ECs could suppress the growth of co-cultured tumor cells. For this purpose, we co-cultured HUVECs and HCCLM3 cells using transwell permeable supports. HUVECs were plated in the inserts, and HCCLM3 cells were plated in the chambers at a 5:1 HUVEC: HCCLM3 cell ratio. The Cell-IQ system was used to monitor the proliferation of the HCCLM3 cells (Fig. 3A). The results showed that the proliferation rate of the HCCLM3 cells negatively correlated with the levels of miR-302c in HUVECs.

We then co-injected HCCLM3 cells and HUVECs (HUVEC, control, miR-302c or anti-302c) into the right flanks of nude mice at a 5:1 HUVEC: HCCLM3 cell ratio. After 4 weeks, all groups formed tumors in the flanks (Fig. 3B). The tumor volume decreased in the miR-302c group, and increased in the anti-302c group (Fig. 3C). These results indicate that the overexpression of miR-302c in ECs can inhibit the growth of co-cultured tumor cells.

### MiR-302c suppresses EndMT through inhibition of MTDH in HUVECs

**Figure 4:**
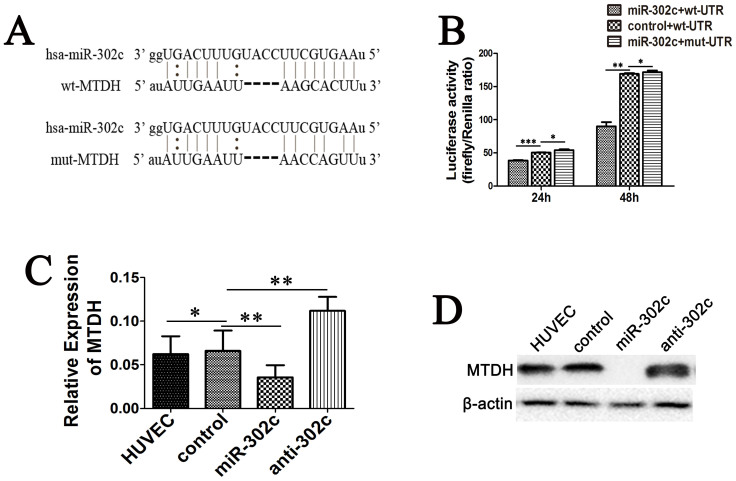
Role of miR-302c in the regulation of MTDH. (A) Potential binding site of miR-302c in the wt- and mut-3′UTR of MTDH mRNA. (B) Reporter gene analysis of the effect of miR-302c on the 3′UTR of MTDH mRNA. (C) Relative mRNA expression of MTDH in HUVECs harboring different levels of miR-302c. (D) Western blot results of MTDH of the cells. (*P > 0.05, **P < 0.01, ***P < 0.05).

To further explore the mechanisms through which miR-302c regulates EndMT in ECs, we next made bio-informative predictions of the potential target genes of miR-302c using the microRNA target predicting program miRanda (http://www.microrna.org/microrna/searchGenes.do). Among the predicted targets, MTDH has two miR-302c binding sites in the 3′UTR of its mRNA (Fig. 4A), and MTDH has been proven to contribute to cell motility^16^,^17^,^18^. Therefore, we hypothesized that miR-302c might inhibit MTDH expression through binding to the 3′UTR of its mRNA and suppressing its translation.

The results of the microRNA target reporter assay showed that up-regulation of miR-302c reduced the luciferase activity of wt-UTR (Fig. 4B); when the binding site of miR-302c in the MTDH mRNA was mutated (Fig. 4A), the luciferase activity did not change with miR-302c overexpression (Fig. 4B). Further RT-PCR (Fig. 4C), western blot (Fig. 4D) and immunofluorescence microscopy (Fig. 2B) analyses also indicated that miR-302c inhibit the expression of MTDH in HUVECs.

**Figure 5:**
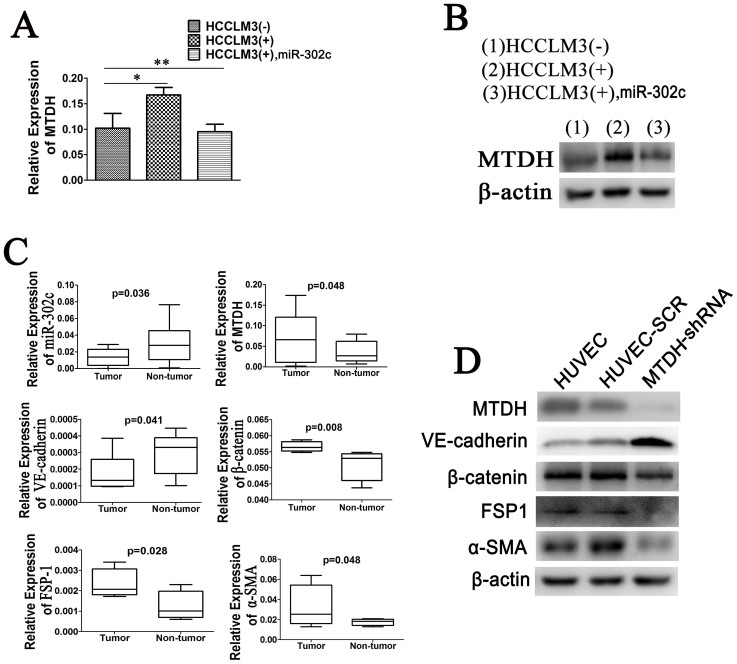
Correlation of miR-302c, MTDH and EndMT. (A) Relative mRNA expression levels of MTDH in HUVECs under different conditions. (B) Western blot results of MTDH in the cells. (C) Comparison of relative levels of miR-302c, MTDH, and EndMT markers in paired tumor and normal ECs from 20 HCC patients using the paired t-test analysis. (D) Western blot analysis of MTDH and EndMT markers in parental, control and MTDH-knockdown cells. (*P < 0.05, **P > 0.05).

Because co-culturing with HCCLM3 cells can lead to a decrease in miR-302c levels in HUVECs, we next investigated whether co-culturing with HCCLM3 cells could enhance MTDH expression in HUVECs via inhibition of miR-302c. RT-PCR (Fig. 5A) and western blot (Fig. 5B) results demonstrated that HUVECs expressed a higher level of MTDH after exposure to HCCLM3 cells. However, when miR-302c was up-regulated in HUVECs, co-culturing with HCCLM3 cells could not increase MTDH expression. We further measured the relative expression of miR-302c, MTDH, and EndMT markers in ECs isolated from tumors and corresponding normal liver tissues of 20 HCC patients by RT-PCR (Fig. 5C). ECs isolated from tumor tissues expressed significantly lower levels of miR-302c and VE-cadherin, but a higher level of MTDH, β-catenin, FSP1 and α-SMA than ECs isolated from normal liver tissues.

Next, we investigated whether the inhibition of EndMT in HUVECs is mediated by MTDH. We stably down-regulated MTDH expression in HUVECs. Western blot results revealed that reduced expression of MTDH in HUVECs led to a higher level of VE-cadherin and lower levels of β-catenin, FSP1 and α-SMA, which can also be observed when EndMT takes place in HUVECs after miR-302c overexpression.

The *in vitro* and *in vivo* data above confirm that miR-302c can suppress MTDH expression through directly binding to the 3′UTR of the MTDH mRNA, thereby inhibiting EndMT in HUVECs.

## Discussion

ECs play key roles in tumor growth and invasion^19^, and the research of tumor-associated ECs can lead us to new strategies for tumor therapy. This study explored miR-302c expression in ECs of HCC tissues and its effect on tumor growth. The results indicated that miR-302c suppresses EndMT of ECs through inhibiting MTDH expression and thus attenuates MTDH's contribution to tumor growth.

MiR-302c was shown to regulate multiple physiological and pathological processes. For instance, Rosa et al.^20^ revealed that the miR-302 family can influence the differentiation of human embryonic stem cells; other studies have indicated that miR-302c directly targets the estrogen receptor in human breast cancer^21^,^22^, and it has also been reported to be dysregulated in biliary tract cancer^23^ and thyroid cancer^24^.

In this study, we proved for the first time that miR-302c in ECs may suppress EC-mediated tumor growth: overexpression of miR-302c in HUVECs markedly decreased the growth of co-cultured HCCLM3 cells both *in vitro* and *in vivo*, and inhibition of miR-302c led to the opposite results. Using samples from 20 HCC patients, we found that the level of miR-302c in ECs isolated from HCC tumor tissues was markedly lower than that from corresponding normal liver tissues. These results imply the importance of miR-302c in tumor-associated ECs.

EndMT is a process whereby ECs lose cell–cell junctions and obtain invasive and migratory properties^6^. The fact that miR-302c can inhibit the motility of ECs and maintain the endothelial morphology of ECs suggested to us that miR-302c might suppress EndMT of ECs. Western blot and immunofluorescence assays validated the intimate correlation between miR-302c and EndMT markers (VE-cadherin, β-catenin, FSP1, and α-SMA). Overexpression of miR-302c in HUVECs resulted in up-regulation of VE-cadherin with down-regulation of β-catenin, FSP1, and α-SMA. VE-cadherin is an EC-specific adhesion molecule that plays an important role in EC biology^25^. VE-cadherin mediates cell–cell junctions in ECs and can affect vascular morphogenesis via Wnt/β-catenin intracellular signaling^26^,^27^. The expression of VE-cadherin and β-catenin is essential for vascular permeability and integrity. FSP1 and α-SMA are both representative mesenchymal markers, and high levels of these two markers imply a mesenchymal phenotype and a deviation from endothelial phenotype^6^. The above results suggest that miR-302c may inhibit EndMT of ECs.

Several studies have proven that ECs can transform into carcinoma-associated fibroblasts (CAFs) through the EndMT process^5^,^6^,^11^. CAFs are a subpopulation of fibroblast-like cells that reside within the tumor microenvironment and promote tumor growth, angiogenesis, inflammation, and metastasis^28^,^29^,^30^. The origin of CAFs is controversial, as several cell types of both mesenchymal and epithelial origins have been considered the source of CAFs, including fibroblasts, smooth muscle cells and ECs^31^. CAF markers are widely described in the literature, among which the most frequently reported to be up-regulated include α-SMA and FSP1^5^. Our results implied that ECs could be an alternative origin of CAFs and that miR-302c could impede the formation of CAFs by obstructing the EndMT of ECs, thereby inhibiting tumor growth. However, whether ECs undergoing EndMT are the sole source of CAFs in HCC will require further investigation.

In this study, we used multiple analyses to validate that miR-302c specifically blocks MTDH expression: a microRNA target reporter assay showed that miR-302c directly contacts the MTDH mRNA through matching sequences in the 3′UTR of MTDH; RT-PCR, western blot and immunofluorescence assays demonstrated that the up-regulation of miR-302c in HUVECs led to a marked down-regulation of MTDH; and data from tumor and normal liver tissues of 20 HCC patients demonstrated that the expression of miR-302c negatively correlated with MTDH expression in isolated ECs.

Recently, MTDH has become considered an important molecule in the modulation of cell proliferation, angiogenesis, drug resistance, and stem cell transformation^32^. Its overexpression is observed in a number of malignancies, including HCC, breast cancer, malignant glioma, and neuroblastoma, and is associated with poor clinical outcome^17^,^18^,^33^,^34^. MTDH has been proven to promote the motility of cancer cells^16^,^17^,^18^. For instance, our former study revealed that MTDH might promote HCC metastasis through the induction of the epithelial-mesenchymal transition (EMT) process^18^. Nevertheless, the function of MTDH in ECs has never been investigated. To our knowledge, our study is the first to reveal that blockage of MTDH expression in HUVECs can inhibit the EndMT process. The changes in EndMT markers after MTDH blockage is similar to the overexpression of miR-302c, which further confirms that miR-302c inhibits EndMT of ECs in hepatocellular carcinoma by directly targeting MTDH.

In conclusion, this study demonstrates that EC-specific miR-302c suppresses tumor growth in HCC through MTDH-mediated inhibition of EndMT. MTDH and miR-302c might provide a new strategy for anti-angiogenic therapy in HCC.

## Methods

### Cells and animals

Animal care and experimental protocols were conducted in accordance with guidelines established by the Shanghai Medical Experimental Animal Care Commission. HCCLM3 (LM3) cells (a highly angiogenic human HCC cell line established at the Liver Cancer Institute, Zhongshan Hospital, Fudan University, Shanghai, China) were maintained in DMEM (GIBCO 11995, Invitrogen, Carlsbad, CA), and HUVECs (8000, Sciencell, Carlsbad, CA) were maintained in EGM-2 medium (CC-4147, Lonza, Switzerland). Co-culturing of LM3 cells and HUVECs was performed as previously described^14^. Male athymic BALB/c nude mice were purchased from the Shanghai Institute of Material Medicine, Chinese Academy of Science, and were raised in specific pathogen-free conditions. Ectopic transplants, and real-time PCR were performed according to the manufacturers' instructions^14^; the employed antibodies are listed in Supplementary Table S1.

### ECs isolation

EC isolation were performed as previously described^14^. Briefly, fresh tumor and corresponding normal liver tissues were made into cell suspension; isolation of ECs from the cell suspension was performed using anti-CD31 antibody coupled to magnetic beads (130-091-935, Miltenyi Biotech, Bergisch Gladbach, Germany) and magnetic cell-sorting using the MACS system (Miltenyi Biotech). To increase the purity of isolated ECs, the cell pellets underwent a second isolation with anti-CD31 beads.

### Cell transfection and clone selection

To overexpress miR-302c, a short hairpin structure against the hsa-miR-302c gene (miR-302c) (F: 5′-TTAAGTGCTTCCATGTTTCAGTGGTTCAAGAGACCACTGAAACATGGAAGCACTTATTTTTTC-3′, R: 5′-TCGAGAAAAAATAAGTGCTTCCATGTTTCAGTGGTCTCTTGAACCACTGAAACATGGAAGCACTTAA-3′) or the negative control (control) (F: 5′-TTTCTCCGAACGTGTCACGTTTCAAGAGAACGTGACACGTTCGGAGAATTTTTTC-3′, R: 5′-TCGAGAAAAAATTCTCCGAACGTGTCACGTTCTCTTGAAACGTGACACGTTCGGAGAAA -3′) was synthesized, annealed, and cloned into the HpaI and XhoI sites of the pSicoR GFP vector, respectively, to construct the shRNA-based lentiviral vectors pSicoR-miR-302c and pSicoR-NC. The vectors were then co-transfected with the packaging plasmids pCMV-VSV-G (0.5 μg) and pCMV-dR8.91 (1 μg) using TransLipid Transfection Reagent (TransGen Biotech, Beijing, China) to construct the viral particles in 293T cells. The infection of HUVECs with the viral particles was performed according to the manufacturer's instructions. The infected cells expressing green fluorescent protein (GFP) were purified by flow cytometry for further experiments. For permanent inhibition of miR-302c, vectors bearing an anti-302c sequence (GCATTAACATGGAATTCCC) were packaged into the virus.

The inhibition of MTDH in HUVECs was conducted using a short hairpin RNA (shRNA)-mediated stable silencing method as previously described^18^.

### Measurement of cell bio-behaviors

The measurement of cell bio-behaviors, including proliferation and cell motility, was carried out using the Cell-IQ system (Chip-man Technologies, Tampere, Finland). Briefly, cells were cultured in a 24-well plate at a density of approximately 2.5 × 10^4^ cells/well. The plate was then placed in the Cell-IQ system and monitored for 4 days. Images were captured and analyzed with the Cell-IQ Analyzer Pro-Write V.AN 2.3.0 (Chip-man Technologies). For cell proliferation, the total cell number was analyzed every 8 hours; for cell motility assays, images were captured at 15 min intervals, and the average moving speed of a single cell (μm/h) was calculated as the motility of the cells, as previously described^35^. Cell migration and scratch assays were performed as previously described^14^.

### MicroRNA Target Reporter Assay

Wild-type (wt-UTR) (F: 5′-CGGGACTAGTCGGGAGGGGAGGTCAGAATAA-3′, R: 5′-GGTCGACGCGTCAGGCAGCTACATCTACTGCTAAAA-3′) and mutant (mut-UTR) (F: 5′-CGGGACTAGTCGGGAGGGGAGGTCAGAATAA-3′, R: 5′-GGTCGACGCGTC AACTGGTTAATTCAATATGACTTC-3′) versions of MTDH mRNA were respectively cloned into the pMIR-REPORT™ microRNA Expression Reporter Vector (AM5795, Applied Biosystems). The 293T cells were transfected with 0.1 μg of firefly luciferase reporter vector containing the target site, 0.1 μg of pSicoR-miR-302c, and 0.01 μg of Renilla luciferase control vector using Lipofectamine 2000 (Invitrogen). Assays were performed at 24 and 48 h after transfection using the dual luciferase reporter assay system (E1910, Promega, Madison, WI). Firefly luciferase activity was normalized to Renilla luciferase activity. The experiments were performed three times in sextuplicates. Relative luciferase activities were calculated from the ratio between firefly and Renilla luciferase activities.

### Statistical analysis

Statistical analyses were performed with SPSS 17.0 software (SPSS, Chicago, IL), as previously described^18^. A two-tailed value of *P* < 0.05 was used to indicate a significant result.

## Author Contributions

J.Z. and K.Z. wrote the main manuscript text; Q.P. and L.Q.J. conducted most microRNA experiments; Z.D. and A.W.K. conducted the measurement of cell bio-behaviors; H.Y.Z. conducted the immunofluorescence assay; Z.Y.T. and J.F. provided professional advices during the whole research. All authors reviewed the manuscript.

## Supplementary Material

Supplementary Information Dataset 1
