# Supplementary material for: MiR-302c inhibits tumor growth of hepatocellular carcinoma by suppressing the endothelial-mesenchymal transition of endothelial cells
Source: Sci Rep. 2014 Jul 16;4:5524. doi: 10.1038/srep05524 (PMC4100019; doi:10.1038/srep05524)
Supplement: Supplementary Information — Dataset 1 [file srep05524-s1.doc]

**MiR-302c inhibits tumor growth of hepatocellular carcinoma by suppressing the endothelial-mesenchymal transition of endothelial cells**

**Kai Zhu^1^,** ***, Qi Pan^1^,** ***, Luo-qi Jia^2^,** ***, Zhi Dai^1^, Ai-wu Ke^1^, Hai-ying Zeng^3^, Zhao-you Tang^1^, Jia Fan^1^, Jian Zhou^1, 4^.**

**Supplementary Tables**

Supplementary table S1. Primers, antibodies and shRNA used in the study

| **Primers for Real-time PCR** | | | | |
| --- | --- | --- | --- | --- |
| **Protein** |  | | **Sequence (5’ 🡪 3’)** | |
| MTDH | F | | AAGTGGCTGAGGGTGAAGCTG | |
|  | R | | CGCTGCTGTCGTTTCTCTCTGT | |
| VE-Cadherin | F | | CAAGCCCTACCAGCCCAAAGTG | |
|  | R | | CCGTGTTATCGTGATTATCCGTGA | |
| β-catenin | F | | ACCAGCCGACACCAAGAAGC | |
|  | R | | GCGGGACAAAGGGCAAGAT | |
| FSP1 | F | | CCAGGAGGATGATCTTCAGCAC | |
|  | R | | TTTAAGCCAGGATGAGGTAGCA | |
| α-SMA | F | | CTCCCTTGAGAAGAGTTACGAGTTG | |
|  | R | | CATGATGCTGTTGTAGGTGGTTTC | |
| β-actin | F | | GCTCCTCCTGAGCGCAAG | |
|  | R | | CATCTGCTGGAAGGTGGACA | |
|  | | | | |
| **Antibodies used in the research** | | | | |
| **Protein** | | **Usage** | | **Antibody** |
| MTDH | | WB, IHC | | ab45338, Abcam |
| β-actin | | WB | | ab92436, Abcam |
| β-catenin | | WB, IF | | ab32572, Abcam |
| α-SMA | | WB | | 1184-1, Epitomics |
| VE-Cadherin | | WB | | BMS158, eBioscience |
| FSP1 | | WB | | 3513-1, Epitomics |

Abbreviations: WB, western blot; IF, immunofluorescence; ELISA, enzyme-linked immunosorbent assay; IHC, immunohistochemistry
